# Intraspecific Differences in Lipid Content of Calanoid Copepods across Fine-Scale Depth Ranges within the Photic Layer

**DOI:** 10.1371/journal.pone.0092935

**Published:** 2014-03-25

**Authors:** Margarita Zarubin, Viviana Farstey, Anette Wold, Stig Falk-Petersen, Amatzia Genin

**Affiliations:** 1 The Interuniversity Institute for Marine Sciences, Eilat, Israel; 2 Department of Ecology, Evolution and Behavior, Silberman Institute of Life Sciences, the Hebrew University of Jerusalem, Jerusalem, Israel; 3 Akvaplan-niva, Fram Centre, Tromsø, Norway; 4 Department of Arctic and Marine Biology, Faculty of Biosciences, Fisheries and Economics, University of Tromsø, Tromsø, Norway; 5 Norwegian Polar Institute, Tromsø, Norway; University of Connecticut, United States of America

## Abstract

Copepods are among the most abundant and diverse groups of mesozooplankton in the world's oceans. Each species has a certain depth range within which different individuals (of the same life stage and sex) are found. Lipids are accumulated in many calanoid copepods for energy storage and reproduction. Lipid content in some species increases with depth, however studies so far focused mostly on temperate and high-latitude seasonal vertically migrating copepods and compared lipid contents among individuals either from coarse layers or between diapausing, deep-dwelling copepods and individuals found in the photic, near-surface layer. Here we examined whether lipid contents of individual calanoid copepods of the same species, life stage/sex differ between finer depth layers within the upper water column of subtropical and Arctic seas. A total of 6 calanoid species were collected from samples taken at precise depths within the photic layer in both cold eutrophic and warm oligotrophic environments using SCUBA diving, MOCNESS and Multinet. Measurements of lipid content were obtained from digitized photographs of the collected individuals. The results revealed significant differences in lipid content across depth differences as small as 12–15 meters for *Mecynocera clausi* C5 and *Ctenocalanus vanus* C5 (Red Sea), *Clausocalanus furcatus* males and two clausocalanid C5s (Mediterranean Sea), and *Calanus glacialis* C5 (Arctic). We suggest two possible explanations for the differences in lipid content with depth on such a fine scale: predator avoidance and buoyancy.

## Introduction

Copepods are among the most abundant metazoans on earth [1]. Many copepods are herbivorous and consequently form an important trophic link between phytoplankton and fish [1]. Most copepods convert part of their food to lipids, stored in their body in an oil sac or as oil droplets for energy storage and reproduction [2]. The lipid content is generally higher in copepods living in cold, high-latitude oceans than in those residing in warmer, tropical and sub-tropical seas [3], [4]. The amount of lipids in a copepod is a function of its feeding and metabolic expenditures in the recent past, and therefore lipid contents vary between individuals belonging to the same species and life stage [5], [6].

It is generally believed that lipids play a role in copepod buoyancy [7]–[11]. This is because lipids are less dense, more compressible and more thermally expandable than seawater [12]. Several studies reported that lipid content in some species increased with depth [3], [6], [10], [13]–[16], sometimes suggesting a role in buoyancy control [10]. However, these studies focused mostly on copepods from temperate and high latitudes exhibiting seasonal vertical migration. Furthermore, the comparisons of lipid contents were so far examined only among coarse depth layers, or between diapausing copepods that dwell in deep waters and those found in the photic, near-surface layer.

Our study was motivated by the common observation that in most species individuals belonging to the same sex and life stage are found across a depth range of the order of 10 s of meters (e.g. [17]). Such ranges might be sufficiently large to induce a difference in body buoyancy relative to water, because the change in lipid density with depth is steeper than that of seawater [7], [18]. Consequently individuals found at different depths should differ in their lipid content. Alternatively, individuals with bigger oil sacs or oil droplets may be more visible to predators and thus may prefer to be in greater, less illuminated depths.

The objective of our study was to refine the depth resolution of existing lipid content comparisons between depths. Therefore, for several calanoid species we examined whether lipid content of individual copepods within a species, sex, and life stage differ between two fine depth layers in the upper water column of subtropical and Arctic seas.

## Materials and Methods

### Ethics statement

The collection of plankton samples in this study was carried out under the guidelines of the Hebrew University Committee for Treatment and Experiments with Animals. Under those regulations, no specific permissions are required for research with plankton. Nor were permits for plankton collection required from the Israeli Nature & Park Authorities and the Norwegian authorities for Fisheries and Coastal Affairs since none of our study sites was located within a nature reserve or involved endangered or protected species.

### Study sites

Copepods were sampled at two oligotrophic, mid-latitude sites: the Gulf of Aqaba (Eilat), Northern Red Sea (29°30' N, 34°55' E) and the Levantine basin of the Eastern Mediterranean Sea off the Israeli coast (32°25' N, 34°49' E), and in a eutrophic Arctic fjord Rijpfjorden in the Svalbard archipelago (80°7' N, 22°9' E).

The Gulf of Aqaba is a desert-enclosed sea. General oceanographic and meteorological conditions are described in [19]. The water temperature ranges from 21°C in February to 28°C in August-September, and conditions in the gulf are generally oligotrophic, with chlorophyll concentrations of 0.05-0.8 μg/L (Israel National Monitoring Program http://www.iui-eilat.ac.il/NMP/Default.aspx). The pelagic zooplankton community is dominated by small copepods <2 mm consisting of a highly diverse assemblage of species [20]–[22].

The Levantine basin of the eastern Mediterranean Sea is an ultra-oligotrophic ecosystem [23], [24] with chlorophyll concentrations in the upper 200 m ranging from 0.01 to 0.4 μg/L, and averaging 0.126 μg/L [24]. The average sea surface temperatures range from ∼17°C in March to ∼27-28°C in August and September (MedAtlas, http://www.ifremer.fr/medar/). The mesozooplankton community is dominated by small copepods [25], [26].

Rijpfjorden is a high arctic fjord on the north coast of the Svalbard archipelago. Arctic fjords are characterized by intense seasonality, reflected in varying stratification. Sea ice plays a central role in controlling the fjord salinity, influencing the exchange with oceanic waters [27]. Rijpfjorden is covered with ice for 6–8 months of the year with low water temperature of approx. −1.8°C [28]. During the sea-ice melt in July-August, the temperature can rise to 3–4°C, and there can be intermittent intrusions of modified Atlantic water into the fjord. A pelagic bloom associated with the sea-ice melt is brief with large inter-annual variations in timing and biomass. Chlorophyll-*a* concentrations at 17 m depth are near zero during most of the year, but peak at 5–10 μg/L in summer [29]. The ice-algae bloom occurring earlier in the season could also be an important food source for herbivorous zooplankton in this fjord [30]. The dominant copepod species and the primary herbivores in the pelagic food web of the Arctic are the large lipid-rich *Calanus finmarchicus*, *C. glacialis* and *C. hyperboreus* ([31], and the references therein).

### Sampling and analysis

Precise depth-stratified samples of zooplankton were obtained using SCUBA diving in the Red Sea and the Mediterranean Sea, a Multiple Opening-Closing Net Environmental Sensing System (MOCNESS) in the Red Sea, and a Multiple Plankton Sampler - Multinet - in the arctic fjord Rijpfjorden. All the samples were taken during daytime except in Rijpfjorden, where zooplankton was sampled also during the night (time of midnight sun).

For a summary of samples taken see Table I. During the SCUBA sampling in the Red Sea two divers towed a plankton net (mesh size 200 μm, mouth opening 0.25 m^2^) at two depths, 5 and 20 m, once a month between May and September 2009 above a bottom depth of 40 m. The SCUBA tows in the Mediterranean Sea were carried out on two consecutive days in September 2011, at 6–7 m and at 20 m depth, above 40 m deep bottom. The MOCNESS (mesh size 100 μm, mouth opening 1 m^2^) was used in the Red Sea for horizontal tows at two depth layers: 20–30 m and 60–70 m, above ∼400 m deep bottom, in August 2010. The Multinet system (mesh size 200 μm, mouth opening 0.25 m^2^) was used in Rijpfjorden in July 2011 for horizontal tows at 8 m and 20 m on 18.7.11 (daytime) and at 6 m and 15 m on 19.7.11 (nighttime; midnight sun conditions), above ∼200 m deep bottom. The difference in depth horizons in Rijpfjorden between day and night was due to logistic reasons. At all sites vertical profiles of temperature and salinity were measured with a CTD (Seabird, SBE9plus profiler). Chlorophyll was extracted from appropriate volumes of seawater that were filtered through 25 mm GF/F filters. The extraction was performed for 20–24 h in the dark at 4°C, either in 90% acetone (Red Sea; data courtesy of the Israel National Monitoring Program http://www.iui-eilat.ac.il/NMP/Default.aspx) or in methanol (Rijpfjorden), and the chlorophyll-*a* concentrations were determined fluorometrically. In the Mediterranean Sea a vertical profile of fluorescence from August 23, 2011, was provided by the Israel Marine Data Center (ISRAMAR) of the Israel Oceanographic and Limnological Research.

**Table 1:** Average prosome lengths (±SD) of all analyzed copepod species at all sites, the results of statistical comparisons and a summary of the samples taken.

Site	Species	Method	Sampling	Date	Depth	n	Mean PL (± SD) (mm)	Statistical test			
Red Sea	*C. vanus*	SCUBA	1	20.5.09	5 m	30	1.18 (±0.057)	Kruskal-Wallis, χ^2^ = 9.122, df = 1, p = 0.003			
	*C. vanus*	SCUBA	1	20.5.09	20 m	30	1.15 (±0.084)				
	*C. vanus*	SCUBA	2	25.7.09	5 m	8	0.83 (±0.046)				
	*C. vanus*	SCUBA	2	25.7.09	20 m	9	1.02 (±0.123)				
	*C. vanus*	SCUBA	3	1.9.09	5 m	30	0.81 (±0.035)				
	*C. vanus*	SCUBA	3	1.9.09	20 m	17	1 (±0.058)				
Red Sea	*M. clausi*	SCUBA	1	25.6.09	5 m	50	1.112 (±0.066)	Kruskal-Wallis, χ^2^ = 2.878, df = 1, p = 0.09			
	*M. clausi*	SCUBA	1	25.6.09	20 m	48	1.113 (±0.036)				
	*M. clausi*	SCUBA	2	1.7.09	5 m	25	1.372 (±0.023)	Kruskal-Wallis, χ^2^ = 2.407, df = 1, p = 0.121			
	*M. clausi*	SCUBA	2	1.7.09	20 m	17	1.365 (±0.023)				
	*M. clausi*	SCUBA	3	1.9.09	5 m	6	1.118 (±0.01)	Kruskal-Wallis, χ^2^ = 0.642, df = 1, p = 0.423			
	*M. clausi*	SCUBA	3	1.9.09	20 m	76	1.11 (±0.024)				
	*M. clausi*	MOCNESS	4	4.8.10	20–30 m	64	0.735 (±0.016)	Kruskal-Wallis, χ^2^ = 13.357, df = 1, p<0.001			
	*M. clausi*	MOCNESS	4	4.8.10	60–70 m	74	0.724 (±0.02)				
Med. Sea	*C. furcatus* male	SCUBA	1	26.9.11	7 m	60	0.621 (± 0.015)	Kruskal-Wallis, χ^2^ = 12.716, df = 1, p<0.001			
	*C. furcatus* male	SCUBA	2	27.9.11	6 m	31	0.634 (±0.016)				
	*C. furcatus* male	SCUBA	1	26.9.11	20 m	42	0.619 (±0.014)				
	*C. furcatus* male	SCUBA	2	27.9.11	20 m	17	0.617 (±0.024)				
Med. Sea	C5 right	SCUBA	1	26.9.11	7 m	48	0.614 (±0.02)	Kruskal-Wallis, χ^2^ = 7.123, df = 1, p = 0.008			
	C5 right	SCUBA	2	27.9.11	6 m	21	0.613 (±0.023)				
	C5 right	SCUBA	1	26.9.11	20 m	42	0.606 (±0.018)				
	C5 right	SCUBA	2	27.9.11	20 m	15	0.598 (±0.022)				
Med. Sea	C5 left	SCUBA	1	26.9.11	7 m	58	0.612 (±0.022)	Kruskal-Wallis, χ^2^ = 13.344, df = 1, p<0.001			
	C5 left	SCUBA	2	27.9.11	6 m	33	0.618 (±0.02)				
	C5 left	SCUBA	1	26.9.11	20 m	59	0.607 (±0.016)				
	C5 left	SCUBA	2	27.9.11	20 m	21	0.602 (±0.01)				
Arctic	*C. glacialis* C5	Multinet		18.7.11, day	8 m	33	3.18 (±0.185)	2-way ANOVA:			
	*C. glacialis* C5	Multinet		19.7.11, night	6 m	23	3.35 (±0.156)	effect of depth: F = 0.000, df = 1, p = 0.988			
	*C. glacialis* C5	Multinet		18.7.11, day	20 m	29	3.22 (±0.167)	effect of stage: F = 749.8, df = 1, p<0.001			
	*C. glacialis* C5	Multinet		19.7.11, night	15 m	28	3.35 (±0.168)				
											
	*C. glacialis* C4	Multinet		19.7.11, night	6 m	20	2.48 (±0.109)				
	*C. glacialis* C4	Multinet		19.7.11, night	15 m	27	2.45 (±0.111)				

Abbreviations: PL – prosome length, SD – standard deviation, Med. Sea – Mediterranean Sea, "C5 right" and "C5 left" are codenames for two clausocalanid copepodites that refer to the position of the longer ramus of the fifth swimming leg.

Sampled copepods were sorted to species [32]–[34] and life stages either on board, immediately after sampling (Arctic), or in the lab up to 2 months later, after being stored at −80°C. For the latter, the collected zooplankton samples were concentrated immediately after sampling to remove excess seawater and rapidly frozen in liquid nitrogen followed by storage at −80°C until sorting. This procedure is recommended when immediate lipid analysis is not possible [35]. When sorting, care was taken to select only undamaged individuals. Each individual was photographed using either a dissecting microscope, for the larger *Calanus glacialis* C5 and C4, or a light microscope for the small *Mecynocera clausi* C5, *Ctenocalanus vanus* C5, *Clausocalanus furcatus* males and two unidentified clausocalanid C5s (hereafter termed "C5 right" and "C5 left", codenames based on the position of the longer ramus of the 5^th^ swimming leg), one of them most probably *C. furcatus* C5. Small copepods that contained a large amount of lipids tended to tilt. Therefore, a special custom-made 300 μm deep depression slide was used to physically prevent tilting of the photographed copepods. Individual copepods were placed in the depression in a drop of seawater, covered with a glass slide, and then digitally photographed. ImageJ software (http://rsbweb.nih.gov/ij/) was used to process the images, by measuring the length and width of the prosome, and the projected area of the oil droplet(s)/oil sac following [36]. Length measurements were calibrated using a grid of known size. The prosome areas of all species and the areas of the oil droplets/sac of *M. clausi* and *C. vanus* were approximated as ellipsoids. For *M. clausi* and *C. vanus* the oil area was calculated based on the diameter of the oil droplet(s) and the length and width of the oil sac, respectively. The projected areas of the oil sacs of *C. furcatus*, the unidentified clausocalanid C5s and *C. glacialis* were calculated after manually digitizing the outer contour of the oil sac. To normalize the lipid content by body size, values of oil sac or oil droplet area are presented as a percentage of the prosome area. A total of 1086 copepods were measured for their lipid content.

### Density calculations

In order to better understand the link between lipid content and copepod density we used a simple model to estimate individual copepod density (7,12). To the best of our knowledge, the density and the pressure-volume-temperature (*PVT*) properties of copepod lipids have been measured only for a lipid mixture, consisting mostly of wax esters, of the subarctic species *Neocalanus plumchrus* ([12], there *Calanus plumchrus*). Warm-water epipelagic copepods have a different lipid composition than high-latitude copepods, in particular they do not accumulate large quantities of wax esters [4], [37]. The physical properties of low-latitude lipid mixtures, namely density, thermal expansion and compressibility, are expected to be different from those of high-latitude copepods. Consequently, calculations of the density of warm-water copepods based on the measured density of a wax-ester mixture and its *PVT*-properties will be inaccurate. Nevertheless, for the sake of a gross assessment, we calculated individual copepod densities at each site and depth assuming that all the species contained an identical wax-ester mixture regardless of sites and depth. Our estimates used the respective temperature profiles of each site and equations (2) and (3) from [7], which are based on the measurements of [12]. A copepod was assumed to consist of three components: water, lipids and 'other tissues' [7], [8]. The volume proportions of these three components summed up to 1. The proportion of 'other tissues' was held constant (0.2), as well as their density (1.08 g/cm^3^; [7]). The volume proportions of lipids were calculated by converting the lipid areas to volumetric values based on the aforementioned geometries of the oil sacs and droplets. The remaining water proportion varied according to the lipid proportion. The density of water inside the copepod was assumed to be the same as the density of the ambient water. This assumption is violated if any of the species is able to use ion replacement as a buoyancy regulation mechanism, as has been found for the antarctic copepod *Calanoides acutus* [38] in the context of diapause. Due to the above assumptions the density estimations, in particular those of the subtropical species should be treated with caution.

### Statistical analyses

For each species permutation-based ANOVA was used to test the effect of depth layer on the lipid content, with sampling date as a covariate (except for *C. glacialis* C4, where only one sampling date was available). To account for multiple comparisons, the p-values were adjusted using the Holm-Bonferroni method [39]. The same analysis was carried out to test the effect of depth layer on the calculated copepod density. For *M. clausi* in the Red Sea, the data from August 2010 were omitted from the statistical analysis because it was the only date that included the 60–70 m depth horizon and did not include the shallow (5 m) depth. The statistical analyses were carried out using R version 3.0.1. [40] with the lmPerm package [41].

Prosome lengths of copepods within a species and life stage were compared between depths using the nonparametric Kruskal-Wallis test due to the inhomogeneity of the variance of both the original and transformed values. This analysis was carried out using SYSTAT V.9.

## Results

### Oceanographic conditions

**Figure 1:**
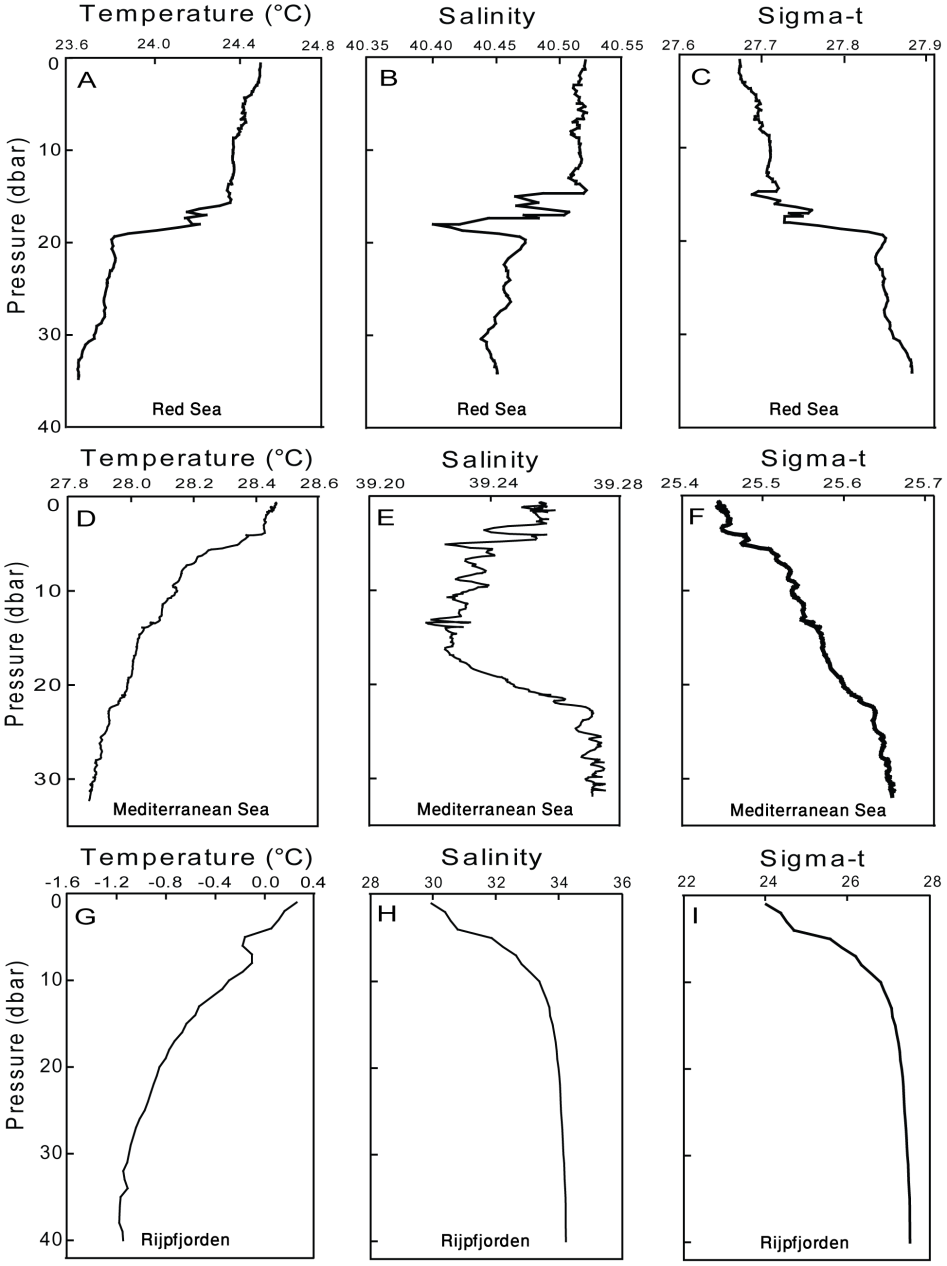
Vertical profiles of seawater temperature (left), salinity (center) and density (right) at each sampling location: A–C: Red Sea (1.7.09), D–F: Eastern Mediterranean Sea (26.9.11) and G–I: Rijpfjorden (18/19.7.12).

**Figure 2:**
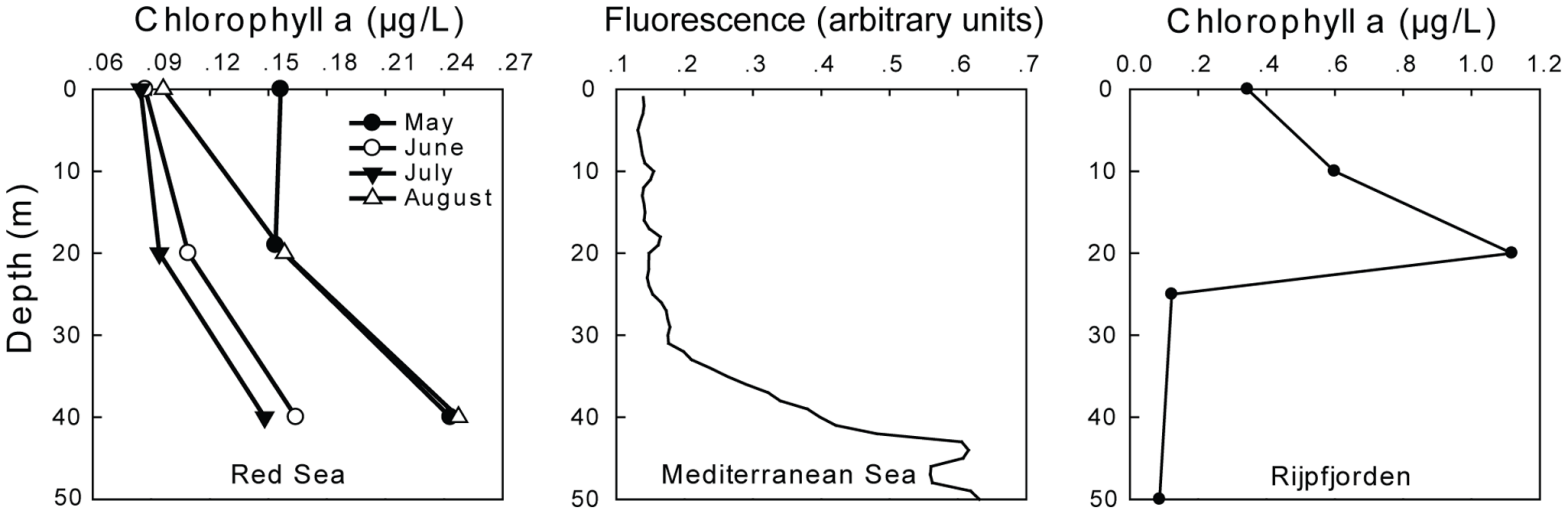
Vertical profiles of Chlorophyll *a* in the Red Sea (left panel) and in Rijpfjorden (right panel), and a fluorescence profile from the Mediterranean Sea (middle panel).

The vertical profiles of seawater temperature, salinity and density at each site during the sampling times are shown in Fig. 1. For the Red Sea the profiles of July 1, 2009 represent the four sampling sessions carried out. The differences in temperature, salinity and density between the depths at which the copepods were sampled were small in the Red Sea and the Mediterranean Sea, and much more pronounced in the Arctic (Fig. 1). In the Red Sea the chlorophyll-*a* concentrations were relatively low (0.085–0.156 μg/L) with similar concentrations found at 5 m and 20 m depths (Fig. 2) except in August 2010, where the concentration at 20 m was slightly higher than at the surface (0.158 μg/L and 0.096 μg/L, respectively; Fig. 2). Similarly, in the Eastern Mediterranean the fluorescence values were very similar at 6–7 m and 20 m (Fig. 2). In Rijpfjorden the chlorophyll-*a* concentration increased from 0.344 μg/L at the surface to 1.119 μg/L at 20 m (Fig. 2).

### Lipid contents versus depth

**Figure 3:**
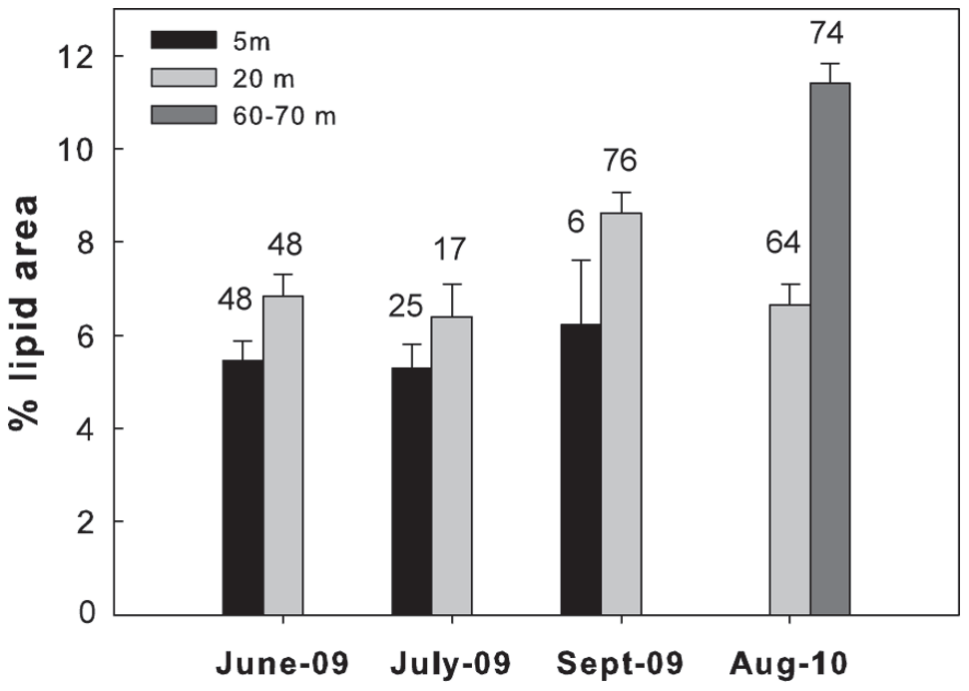
Average lipid content of *Mecynocera clausi* C5 from the Gulf of Aqaba, Red Sea, at 5 m (black bars), 20 m (light grey bars) and 60–70 m (dark grey bar), expressed in % lipid area. Error bars indicate standard error and the numbers above the bars indicate the sample size.

**Figure 4:**
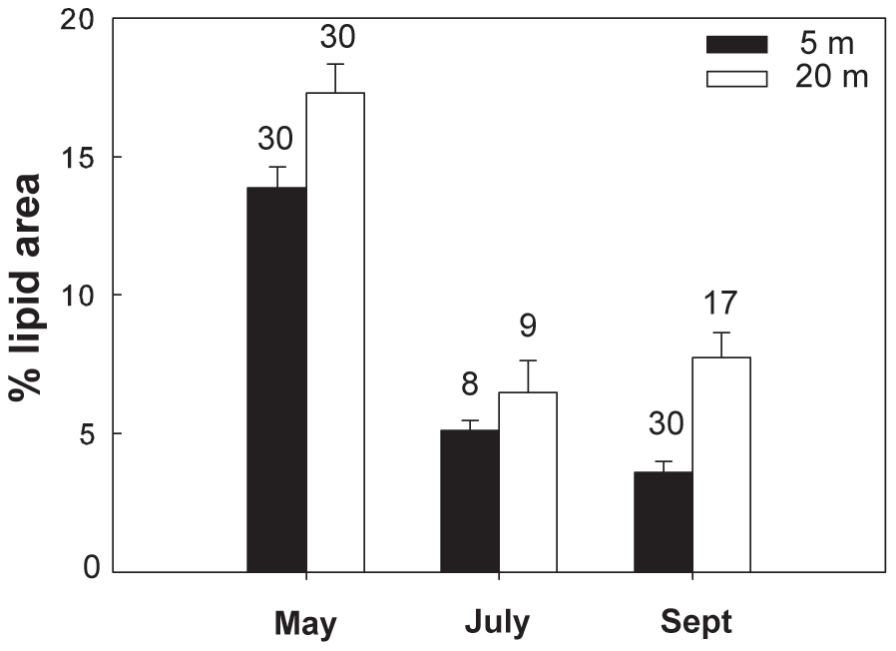
Average lipid content of *Ctenocalanus vanus* C5 from the Gulf of Aqaba, Red Sea, at 5 m (full bars) and 20 m (open bars), expressed in % lipid area. Error bars indicate standard error and the numbers above the bars indicate the sample size.

**Figure 5:**
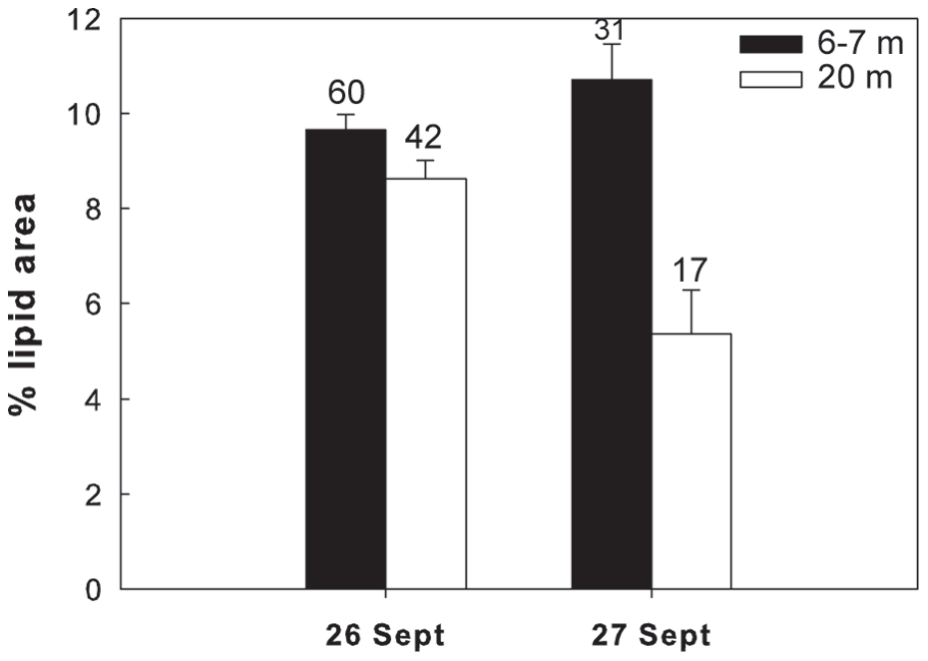
Average lipid content, expressed in % lipid area, of *Clausocalanus furcatus* males at 6–7 m (full bars) and 20 m (open bars). Error bars indicate standard error and the numbers above the bars indicate the sample size.

**Figure 6:**
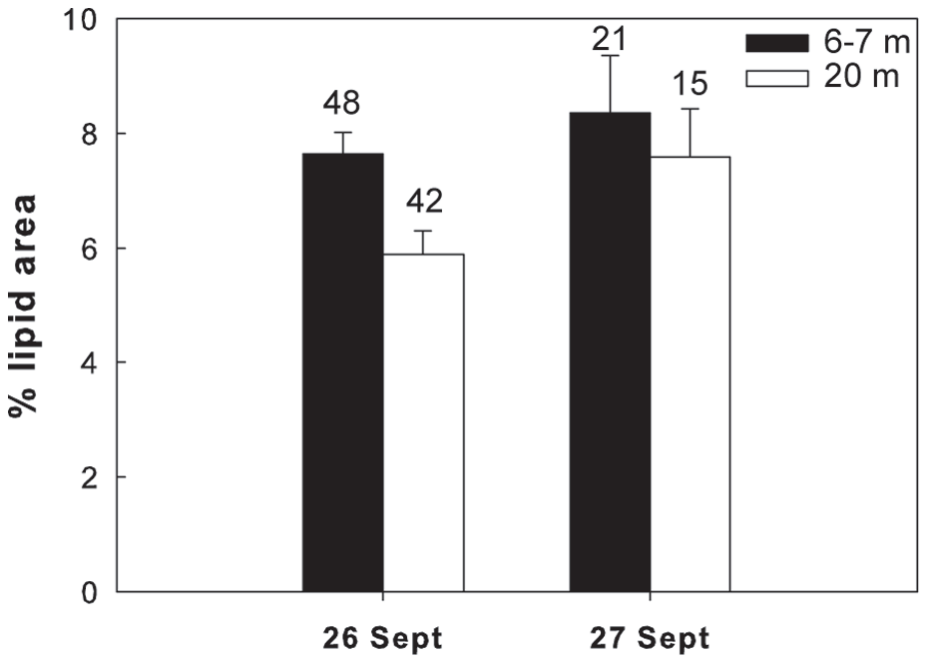
Average lipid content, expressed in % lipid area, of an unidentified clausocalanid C5 “C5 right” from the Eastern Mediterranean Sea at 6–7 m (full bars) and 20 m (open bars). “C5 right” is a codename that refers to the position of the longer ramus of the fifth swimming leg. Error bars indicate standard error and the numbers above the bars indicate the sample size.

**Figure 7:**
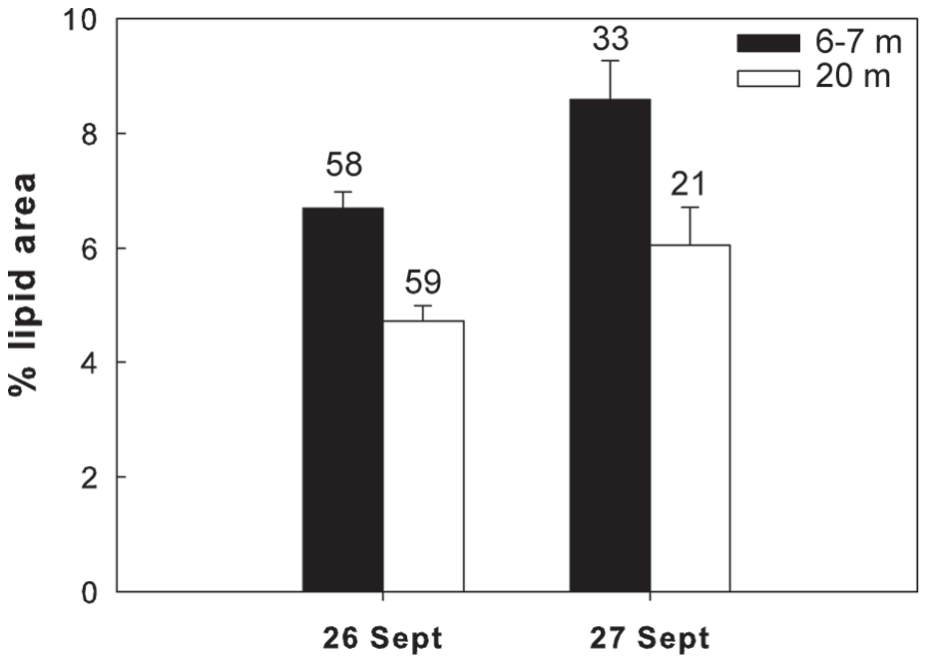
Average lipid content, expressed in % lipid area, of an unidentified clausocalanid C5 “C5 left” from the Eastern Mediterranean Sea at 6–7 m (full bars) and 20 m (open bars). “C5 left” is a codename that refers to the position of the longer ramus of the fifth swimming leg. Error bars indicate standard error and the numbers above the bars indicate the sample size.

**Figure 8:**
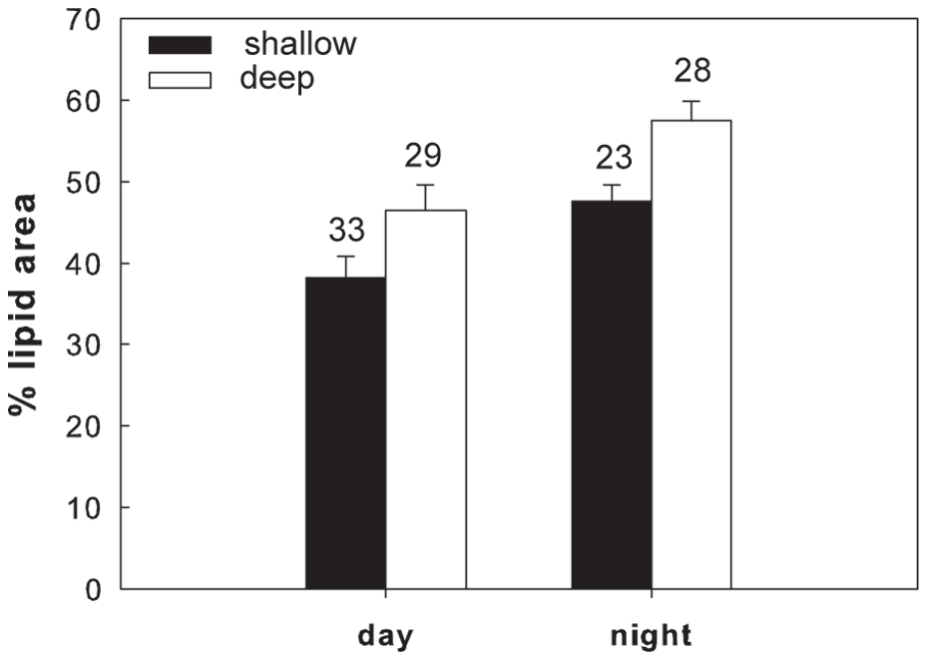
Average lipid content of *Calanus glacialis* C5, expressed in % lipid area, in the Arctic fjord Rijpfjorden at shallow (full bars) and deep (open bars) depth horizons (please see text for details on the sampling depths). Error bars indicate standard error and the numbers above the bars indicate the sample size.

**Figure 9:**
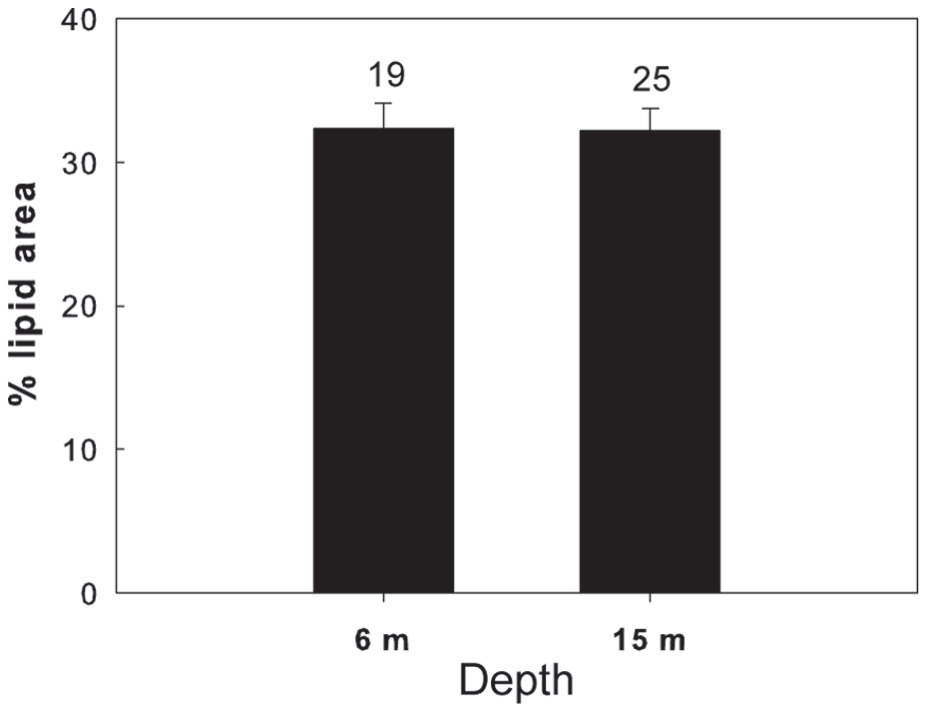
Average lipid content, expressed in % lipid area, of *Calanus glacialis* C4 in the Arctic fjord Rijpfjorden at 6 m (full bars) and 15 m (open bars). Error bars indicate standard error and the numbers above the bars indicate the sample size.

Overall in 6 of the 7 different species/life stages examined at the three sites, the lipid content of the shallower and deeper individuals differed significantly. In the Red Sea, a significantly higher lipid content was found in the deeper individuals of *M. clausi* C5 (Fig. 3; for statistical details see Table II). Similarly, the lipid content of *C. vanus* C5 at that site was significantly higher at 20 m than at 5 m (Fig. 4, Table II). The lipid content was significantly different among depths also in the Mediterranean copepods *C. furcatus* males and the clausocalanids "C5 right" and "C5 left" (Fig. 5–7, Table II). However, the trend here was reversed, with the shallower copepods having more lipids (Fig. 5–7). The lipid contents of the Arctic *C. glacialis* C5 are shown in Fig. 8. Significantly higher lipid contents were found in deeper individuals of *C. glacialis* C5, whereas the lipid contents did not significantly differ between depths in *C. glacialis* C4 (Fig. 9, Table II).

**Table 2:** Summary of the results of the permutation-based ANOVA testing, for each species, the effect of depth layer on the copepod % lipid area, with sampling date as a covariate.

Species	df	Iterations	adjusted p-value
*M. clausi*	1	5000	<0.005
*C. vanus*	1	5000	<0.0001
*C. furcatus*	1	5000	<0.0001
C5 right	1	5000	<0.025
C5 left	1	5000	<0.0001
*C. glacialis* C5	1	5000	<0.0001
*C. glacialis* C4	1	161	NS

To account for multiple comparisons the p-values were adjusted using the Holm-Bonferroni method.

**Figure 10:**
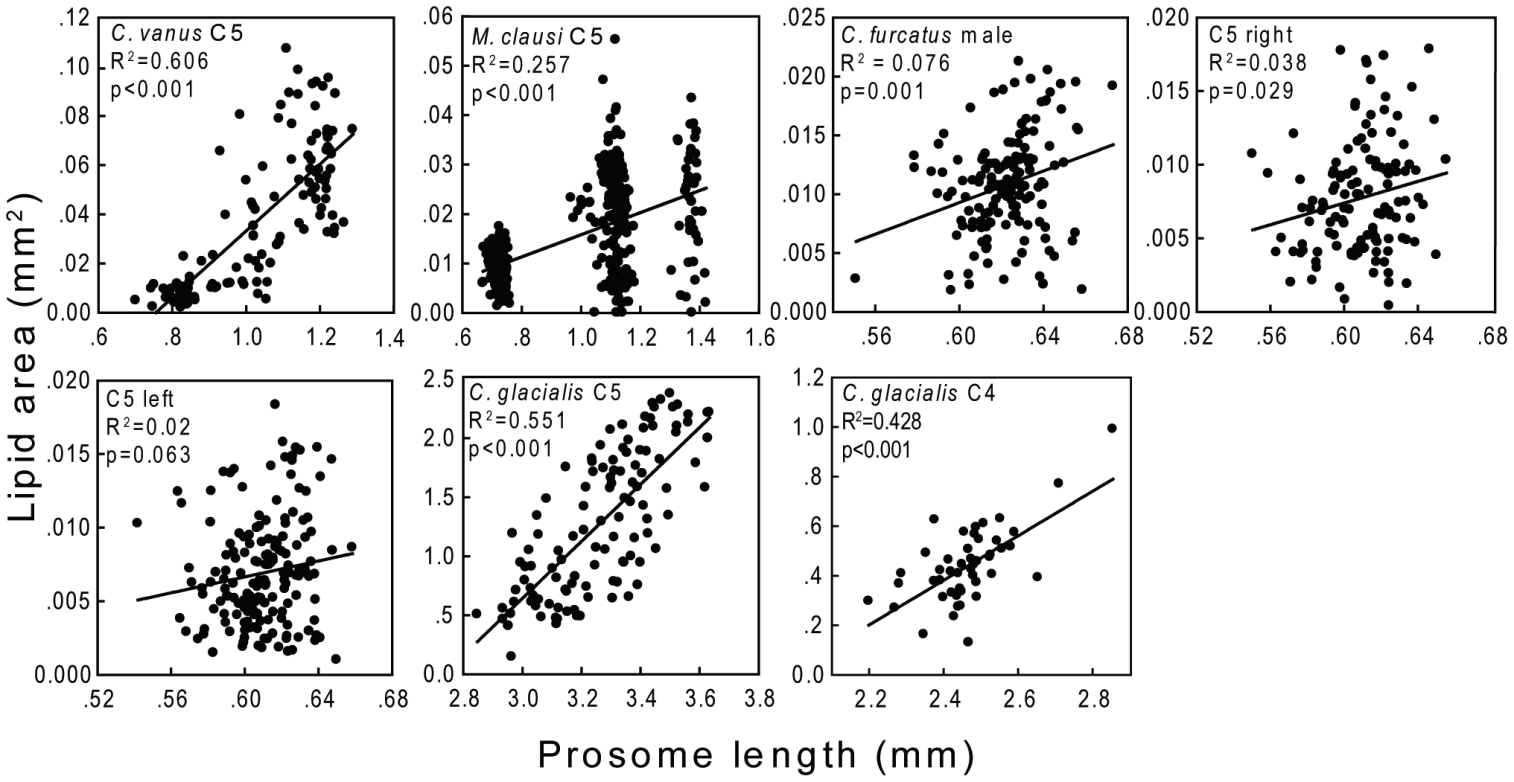
Relationships between prosome length and absolute lipid area for each species.

We scaled lipid content by copepod size by using the parameter percent lipid area. This assumes that there is a relationship between both variables, the body size and the lipid content. In all the species except the clausocalanid “C5 left” the relationship between prosome length and absolute lipid area was significant, however in some cases the R^2^ values were low (Fig. 10). The use of ratios to scale experimental data can lead to spurious results, since the characteristics of the variance of ratios are unpredictable [42], [43]. Therefore, in addition to percent lipid area we ran the statistical analysis without the scaling by testing the effect of depth layer on the absolute value of lipid area (instead of % lipid area). For all the copepod species/stages the results were statistically significant with significance values being similar to those of the scaled data, indicating that the results obtained for the scaled values are not spurious.

### Copepod density

The results of the calculated copepod density are summarized in Table III. In all the copepod species/stages except of *M. clausi* and *C. glacialis* C4 the density differed significantly with depth (Table IV). In *C. vanus* and *C. glacialis* C5 the deeper copepods were significantly less dense than the shallower ones (Tables III–IV), whereas in *C. furcatus* males and the copepodits “C5 right” and “C5 left” the deeper individuals were denser than the shallower ones (Tables III–IV). As mentioned above, these results should be treated with caution due to the assumptions involved (see Methods).

**Table 3:** Summary of the calculated copepod densities for each species, sampling date and depth.

Species	Month/date	Depth (m)	Mean copepod density (±SD) (g/cm^3^)
*M. clausi*	Jun-09	5	1.0302 (±0.00413)
	Jun-09	20	1.0306 (±0.00444)
	Jul-09	5	1.0327 (±0.00319)
	Jul-09	20	1.0313 (±0.00402)
	Aug-10	20–30	1.0269 (±0.01122)
	Aug-10	60–70	1.0119 (±0.01622)
	Sept-09	5	1.0278 (±0.00666)
	Sept-09	20	1.0155 (±0.02138)
*C. vanus*	May-09	5	1.0303 (±0.00301)
	May-09	20	1.0234 (±0.00731)
	Jul-09	5	1.0342 (±0.00115)
	Jul-09	20	1.0336 (±0.00290)
	Sept-09	5	1.0353 (±0.00209)
	Sept-09	20	1.0316 (±0.00397)
*C. furcatus*	26.9.11	7	1.0294 (±0.00218)
	26.9.11	20	1.0303 (±0.00189)
	27.9.11	6	1.0281 (±0.00372)
	27.9.11	20	1.0325 (±0.00298)
C5 right	26.9.11	7	1.0310 (±0.00193)
	26.9.11	20	1.0324 (±0.00189)
	27.9.11	6	1.0324 (±0.00382)
	27.9.11	20	1.0311 (±0.00257)
C5 left	26.9.11	7	1.0318 (±0.00164)
	26.9.11	20	1.0332 (±0.00140)
	27.9.11	6	1.0301 (±0.00292)
	27.9.11	20	1.0322 (±0.00228)
*C. glacialis C5*	18.7.11	8	1.0193 (±0.01384)
	18.7.11	20	1.0123 (±0.01680)
	19.7.11	6	1.0126 (±0.00907)
	19.7.11	15	1.0022 (±0.01220)
*C. glacialis C4*	19.7.11	6	1.0233 (±0.00397)
	19.7.11	15	1.0252 (±0.00414)

**Table 4:** Summary of the results of the permutation-based ANOVA testing, for each species, the effect of depth layer on the copepod density, with sampling date as a covariate.

Species	df	Iterations	adjusted p-value
*M. clausi*	1	1888	NS
*C. vanus*	1	5000	<0.0001
*C. furcatus*	1	5000	<0.0001
C5 right	1	5000	<0.02
C5 left	1	5000	<0.0001
*C. glacialis* C5	1	5000	<0.001
*C. glacialis* C4	1	265	NS

To account for multiple comparisons the p-values were adjusted using the Holm-Bonferroni method.

### Relationships between prosome length and depth

Average prosome lengths for each species at each depth and each study site are summarized in Table I. In *C. glacialis* C5 and C4 and in *M. clausi* C5 there were no significant differences in prosome length between depths, except that the *M. clausi* individuals from the MOCNESS sample from 20–30 m were larger than those from 60–70 m (for statistical details see Table I). Due to the substantial differences of *M. clausi* prosome length in the different months, the analysis of its prosome length relationship with depth was performed separately for each sampling date. In *C. vanus* C5, *C. furcatus* males and the two clausocalanid C5s, significant differences in prosome length were found between different depths. In the Red Sea *C. vanus* C5 specimens were larger at 20 m than at 5 m, except for the first sample where the trend was opposite (Table I). In contrast, *C. furcatus* males and the clausocalanids "C5 right" and "C5 left" in the Mediterranean Sea were larger at 6–7 m than at 20 m.

## Discussion

This study shows, for the first time, differences in lipid content among individuals found at finely segregated depths within the photic layer in several calanoid copepod species. While differences across much coarser depth ranges are well documented [3], [6], [10], [13]–[16], the fine spatial scale of the segregation documented here is surprising. Why would lipid content and copepod body density differ on this spatial scale? We suggest two possible explanations: predator avoidance and buoyancy control.

### Predator avoidance

Many marine predators search for prey visually, and their success in finding it is directly linked to ambient irradiance. Planktonic organisms can lower the risk of visual predation by moving to greater depth. This adaptive behavior is related to body size, i.e. the larger and more visible zooplankters are found at greater depths [44]. In the diel vertical migrator *Metridia pacifica* there was a depth segregation according to body size and lipid sac size in the upper 175 m [15]. The authors suggested that the individuals with larger lipid sacs did not rise into the surface waters at night to minimize predation risk. In our study *M. clausi* and *C. vanus* from the Red Sea, and *C. glacialis* C5 from the Arctic had larger lipid contents at 20 m compared to 5 m. However, none of these species, except of *C. vanus* on a single sampling date, exhibited larger body size at depth. Furthermore, in *C. furcatus* and the two clausocalanid C5s the shallower individuals were significantly larger than the deeper ones. Thus, the majority of our findings does not support the predator avoidance hypothesis, unless the lipid content affects the visibility of the individual more that its body size - a yet untested hypothesis.

### Buoyancy

Differences in lipid content between coarse depth layers or between deeply diapausing stages and active stages from the photic layer have been previously documented in the literature [3], [6], [10], [13]–[16], sometimes in relation to buoyancy control [10], as lipids are less dense, more compressible and more thermally expandable than seawater [12]. In our study we observed that in several species a higher lipid content is found at 20 m than at 5 m. Calculating the individual density showed that the deeper specimens were less dense than the shallower ones. Is it possible that copepods with larger lipid contents prefer to reside in deeper, cooler water to compensate for their excess buoyancy? By influencing the overall buoyancy of the animal lipid content can be related to metabolic expenditure of copepods, e.g. [8] suggested that lipid stores and the resulting buoyancy changes can assist copepods in ascent/descent during seasonal vertical migrations. Density and thus buoyancy also play a role in feeding in feeding-currents creating copepods [45], [18]. Most copepods are denser than seawater [1], and it has been suggested that this excess density is the "anchor" that allows copepods to generate effective feeding currents [45]. Hence a change in body density is expected to affect the pattern and intensity of its feeding currents [18]. To keep a desired density difference relative to seawater a copepod should either digest some of the accumulated lipids or swim to greater depths where due to differential compressibility the excess lipid would be less buoyant. Once at a desired depth, the animal would stay there until its state of buoyancy has changed. In fact, a recent report [46] showed that zooplankters, mostly copepods, retained their depth by swimming against downwelling and upwelling currents. We suggest that actively choosing a specific depth according to individual lipid content could be a buoyancy compensation mechanism assisting individual copepods to retain their normal feeding efficiency.

In the three clausocalanid copepods from the Mediterranean Sea - *C. furcatus*, "C5 right" and "C5 left" - a reverse trend was found: deeper copepods had less lipids and were denser than shallower individuals. Interestingly, while most calanoid copepods generate feeding currents [1], [47], [48], *C. furcatus* does not appear to do so [49]. If this is also true for the two clausocalanids “C5 right” and “C5 left”, the observation of [49] together with our results indirectly suggest a linkage between lipid content, feeding mode (currents or no currents), and depth.

Note however, that other inter-specific differences may also contribute to depth selection in copepods. Although the different observed trends do not refute the hypothesis that lipid content influences the position of a copepod in the water column, it points out that the depth of an individual copepod is likely a product of a complex interplay among several internal and external factors.

Different individuals belonging to the same species and life stage are commonly dispersed across a substantial depth range sometimes reaching tens of meters (e.g. [17]). Usually this depth range is referred to as a feature of the population, and to the best of our knowledge there are no studies showing active depth selection by individual copepods within that range. The vertical distribution of copepods is governed by a number of factors including light, salinity, temperature [50]–[52], oxygen [e.g. 53], food demand [54], food availability [e.g. 55,56], predation risk [57], bottom depth and water clarity [58], turbulence [59], [60], and vertical mixing [61], [62]. Pressure has also been suggested to be a possible factor regulating the vertical distribution of zooplankton [63]. Our findings suggest that lipid content might be an additional factor determining the vertical position of a copepod within the photic layer.

In agreement with past studies, our study shows that epipelagic copepods from low-latitude oligotrophic oceans contain relatively small amounts of lipids [52]. However, the occurrence of fine-scale depth segregation according to lipid content in small, subtropical copepods questions the generally accepted perception that lipid content is not a key ecologically important factor in warm-water copepods [4]. On the contrary, our study suggests that lipid content is likely an important biological factor also in copepods of oligotrophic, low-latitude oceans.

This study demonstrates the importance of an individual-based approach when examining vertical distributions of copepods and emphasizes the need for further studies on the role of lipids in fine-scale regulation of buoyancy in zooplankton.
